# Quality of end-of-life care among individuals with and without dementia: a Swedish registry-based study

**DOI:** 10.1186/s12904-026-02037-9

**Published:** 2026-03-07

**Authors:** Lisa Kastbom, Johan Lyth, Johanna Simmons, Lisa Martinsson, Maria Eriksdotter, Staffan Lundström, Katarina Nägga, Anna Segernäs

**Affiliations:** 1 Department of Health, Medicine and Caring Sciences, Linköping University, Linköping, Sweden; 2 Primary Healthcare Center Ekholmen, Region Östergötland, Linköping, Sweden; 3 Clinical Department of Geriatrics and Palliative Medicine in Linköping, Region Östergötland, Linköping, Sweden; 4 Department of Clinical Sciences, Unit of Professional Development, Umeå University, Umeå, Sweden; 5 Division of Clinical Geriatrics, Department of Neurobiology, Care Sciences and Society, Karolinska Institutet, Stockholm, Sweden; 6 Theme Inflammation and Aging, Karolinska University Hospital, Huddinge, Sweden; 7 Department of Research and Development, Stockholms Sjukhem Foundation, Stockholm, Sweden; 8 Department of Oncology-Pathology, Karolinska Institutet, Stockholm, Sweden

**Keywords:** End-of-life care, Palliative care, Dementia, Place of death, Advance care planning, Quality of end-of-life, Quality registry

## Abstract

**Background:**

Despite dementia being a leading cause of death and clinical guidelines recommending palliative care, substantial gaps in care quality for this population have previously been shown. This study aimed to investigate and compare the quality of end-of-life (EOL) care provided to individuals with and without dementia in different settings.

**Methods:**

In this registry-based study, patients registered in the Swedish Register of Palliative Care (SRPC) between 2011 and 2020 were cross-referenced with patients in the Swedish registry for cognitive/dementia disorders (SveDem). For each patient with dementia registered in SveDem (*n* = 39 712), two controls without dementia matched by year of birth and gender were selected from the SRPC (*n* = 79 336). Quality indicators in the SRPC were analyzed by group (dementia/controls) and by place of death, separately, using the chi-squared test. Multiple logistic regression analyses were conducted to examine the association between the quality indicators and having a diagnosis of dementia or not, overall and in different settings.

**Results:**

Individuals with dementia were more likely to have staff or family members present at death, to receive documented decisions to shift to EOL care, have symptom assessments made the final week of life and prescription of injectables. However, they were less likely to express preferences for place of death and to be informed about EOL care transitions, especially in hospitals without specialized palliative care.

**Conclusions:**

In this study, individuals with dementia received higher quality EOL care in several domains compared with those without dementia. However, they were less likely to have expressed preferences for place of death. These findings highlight the need for early, proactive care planning to align care with patient preferences and avoid potentially non-beneficial actions.

**Supplementary Information:**

The online version contains supplementary material available at 10.1186/s12904-026-02037-9.

## Background

Traditionally, palliative care has focused on individuals with cancer [1]. Over the years, the perspective has broadened to also include non-malignant conditions, such as dementia [2–7]. Dementia is an escalating global challenge, particularly as the population ages. It is currently the seventh leading cause of death and one of the major causes of disability and dependency among older people [8]. As a leading cause of death and an incurable, progressive condition, dementia necessitates end-of-life (EOL) care planning as a core aspect of care [9, 10]. In line with this, the British NICE (National Institute for Health and Care Excellence) guidelines on dementia, aimed at patients, families, and healthcare professionals, include a dedicated section emphasizing the importance of palliative care for individuals with dementia [11]. However, despite existing recommendations, previous studies have identified deficiencies in the quality and consistency of palliative care provided to this patient group. For example, EOL care for individuals with dementia is often inadequately planned, which can result in avoidable hospital admissions and insufficient support for families [12–14]. Family members of patients with dementia are often dissatisfied with the level of communication and their involvement in the decision-making processes [15].

While the dying experience is highly individual, there is broad consensus that the quality of EOL care can be defined across a set of key domains: physical comfort, psychological and spiritual well-being, shared decision-making, communication and information, family support and access to care [16, 17]. A prerequisite for providing good palliative care is the ability to identify patients who are approaching the end of their lives. In Sweden, there are approximately 90 000 deaths each year, the majority (80%) of which are anticipated deaths [18]. This means that in most cases, there are good opportunities to make proactive plans regarding the content and direction of care for patients approaching EOL. However, it remains challenging to identify an appropriate time to initiate palliative care in patients with dementia [19–21].

Nursing homes (NHs) are common places of death for older people [22, 23] and particularly for individuals with dementia [24–27]. Previous research has demonstrated that patients with dementia tend to receive higher-quality EOL care in NHs compared to hospital settings. In a study by Martinsson et al., hospital deaths were associated with lower quality of EOL care across 10 out of 13 measured quality indicators compared to deaths occurring in NHs [4]. However, considerable variation in the degree of symptom relief during the final week of life among older people also in NHs highlights the need to improve palliative care, with particular emphasis on symptom management and EOL communication [28]. Additionally, an increasing number of individuals suffering from dementia remain in their ordinary homes, and the global movement of encouraging aging in place reinforces this trend [29]. To ensure a good quality of EOL care for patients and their families in ordinary homes, well-resourced community-based support, including home care and management of complex conditions in primary care, are needed [30].

Recommendations state that hospital care for people with dementia should be carefully considered in relation to care goals and the stage of the disease [31]. Despite this, hospital care and life-sustaining treatments at hospitals still often occur, also at late stages of the disorder [32]. Terminally ill patients admitted to hospital with non-cancer conditions, such as dementia, may thus miss out on opportunities for specialized palliative care [33].

There is a lack of studies comparing the quality of EOL care between individuals with and without dementia, also in relation to place of death. Therefore, the overall aim of this study was to investigate and compare the quality of EOL care provided to individuals with and without dementia in different settings, acknowledging that data on dementia severity were not available. In this manuscript, the term “ordinary homes” refers to private residences, not NHs.

## Methods

### Aim

The aim of this study was to investigate and compare the quality of EOL care provided to individuals with and without dementia in different settings, acknowledging that data on dementia severity were not available.

### Design and setting

The design of this study was explorative and registry-based. Data were obtained through linkage between two national quality registries, the Swedish Register of Palliative Care (SRPC) and the Swedish registry for cognitive/dementia disorders (SveDem), based on unique personal identification numbers. Linkage was performed by one of the two involved registry centers, the Uppsala Clinical Research center (UCR) [34].

### Registries and variables

#### The Swedish register of palliative care

The SRPC collects data on the quality of EOL care in Sweden using a 27-item questionnaire [35, 36], which was originally developed based on the principles of a “good death” as defined in the guidelines of the British Geriatrics Society [37]. Data on care delivered during the last week of life is completed by healthcare staff filling out a web-based questionnaire retrospectively after the patient’s death. Symptoms are assessed based on personal knowledge of the patient and, when present, on documented assessments in the patient’s medical records (e.g. Visual Analogue Scale (VAS) and the Numeric Rating Scale (NRS), which are both validated pain intensity measures, and the Abbey Pain Scale, developed for individuals with end- or late-stage dementia who were unable to articulate their needs). Cause of death data are collected from the SRPC and are regularly linked with the Cause of Death registry at the National Board of Health and Welfare. Of all deaths in Sweden, on average 63% were registered in the SRPC during the study period [38].

SRPC variables used in this study included place of death and expressed preferences regarding place of death, if the death was anticipated or not, causes of death, presence of staff and/or family members at the moment of death, whether transition to EOL care was documented in patient records, documented EOL discussions with the patient and family members, time since last physician examination, symptom prevalence, and prescription of injectables for symptom relief (pain, anxiety, nausea and rattles).

#### The Swedish registry for cognitive/dementia disorders (SveDem)

This register’s aim is to enhance diagnostic and care quality of patients with cognitive/dementia disorders. To date, the registry includes more than 141 000 unique individuals, thus providing a database [39] with clinically characterized dementia diagnoses [40]. The variables obtained from SveDem and utilized in this study included: age at baseline registration, gender and type of dementia diagnosis.

### Study population

All patients registered in the SRPC between 2011 and 2020 were cross-referenced with all patients who had a date of death during the same period in SveDem. The study initially identified 39 737 patients with dementia. Each of these patients was matched with two controls from the SRPC (*n* = 79 474). Following the exclusion of 163 cases (25 patients with dementia and 138 controls) due to missing information on place of death, the final sample comprised 39 712 dementia cases and 79 336 controls. The matching was based on year of birth and gender, including only those who did not have a dementia diagnosis according to the International Classification of Diseases (ICD-10) codes F00, F01, F02, F03, G30, or G31, and who did not have dementia listed as the cause of death in the SRPC, to create a control group to contrast with our well-characterized dementia participants. To enhance statistical efficiency and internal validity, a 2:1 matching ratio was applied. Using two controls per case provides a meaningful increase in statistical power, compared to using only one control. This approach also allows for a more efficient use of the extensive registry data and contributes to more stable and robust risk estimates by reducing the influence of atypical values within the control group. We did not consider it justified to use more than two controls, given the already large size of the dataset. The dataset was pseudonymized before delivery to the researchers.

### Statistical analysis

Descriptive statistics were used to characterise patients with a diagnosis of dementia and matched controls. Quality indicators in the SRPC (see above) were analyzed by group (dementia/controls) and by place of death, separately, using the chi-squared test. Multiple logistic regression analyses were conducted to examine the association between ten quality indicators: (1) anticipated death (2), expressed preferences of place of death (3), someone present at the moment of death (4), time since the last physician examination (5), documented decision to shift to EOL care (6), patient informed about transition to EOL care (7), family member(s) given information about transition to EOL care (8), pain assessed and documented during last week of life (9), symptoms other than pain assessed and documented during last week of life, and (10) prescription of pro re nata (PRN) drugs against pain, anxiety, nausea and rattles, and having a diagnosis of dementia or not, overall and across different settings. Separate models for each indicator were conducted, and all models were adjusted for gender, year of birth, and place of death. Adjusted odds ratios with 95% confidence intervals were illustrated in a forest plot. For each indicator and category, average marginal effects (AMEs) with 95% confidence intervals were additionally calculated and, for interpretability, multiplied by 100 to express the results in percentage-point units.

Statistical analyses were performed using R version 4.5.2 and IBM SPSS Statistics version 29.0.

## Results

### Demographics

Mean age at baseline registration in SveDem was 81 years (range 39–105) and approximately 57% were women. The most common dementia diagnoses were Alzheimer disease and non-specified dementia, followed by vascular dementia and mixed dementia. Descriptive characteristics of the study sample are presented in Table 1.

**Table 1:** Characteristics of the study sample

	Dementia group *n* 39 712		Control group *n* 79 336	
	*n*	%	*n*	%
Age at baseline registration in SveDem (years)	39 – 105 (mean 81)			
Gender				
- Female	22683	57.1	22683	57.1
- Male	17029	42.9	17029	42.9
Age at death (years; mean, (SD))	85 (7)		84 (7)	
Time from dementia diagnosis until death (years)	0 – 13.89 (mean 3.85)			
Dementia diagnosis				
- Mixed dementia	7281	18.3		
- Dementia, non-specified	10383	26.1		
- Alzheimer disease, late onset	10211	25.7		
- Alzheimer disease, early onset	887	2.2		
- Dementia in - Parkinson’s disease	691	1.7		
- Frontotemporal dementia	569	1.4		
- Lewy body disease	931	2.3		
- Vascular dementia (subcortical vascular dementia included)	7817	19.7		
- Other	938	2.4		
- Missing data	4	0.0		

### Place and cause of death

Table 2 illustrates detailed information on place and causes of death. When categorized by place of death, 77% of patients with dementia died in NHs, compared to 34% in the control group. In contrast, hospital deaths without specialized palliative care were less common among patients with dementia (18%) than in the controls (40%). A smaller proportion of patients with dementia died in ordinary homes without specialized palliative care (3%) compared to 8% in the control group, and in specialized palliative inpatient units (2%) compared to 12% in the control group. Deaths in ordinary homes with access to specialized palliative care were rare in both groups, accounting for less than 1% among patients with dementia and 6% in the control group.

**Table 2:** Place and cause of death in the study sample

Reported place of death in SRPC	Total *n* 119 048		Dementia group *n* 39 712		Control group *n* 79 336		*p-*value
	*n*	%	*n*	%	*n*	%	<0.001
Ordinary home, with specialized palliative home care	5003	4.2	267	0.7	4736	6.0	
Ordinary home, without specialized palliative home care	7240	6.1	1095	2.8	6145	7.7	
Specialized palliative inpatient care	10145	8.5	852	2.1	9293	11.7	
Inpatient care, not specialized palliative care	38953	32.7	7003	17.6	31950	40.3	
Nursing home, short term homes included	57707	48.5	30495	76.8	27212	34.3	
**Reported cause of death in SRPC**							
Cancer diseases	34590	29.1	4481	11.3	30109	38.0	<0.001
Circulatory diseases/cardiovascular diseases	37777	31.7	11565	29.1	26212	33.0	<0.001
Respiratory diseases	12547	10.5	3071	7.7	9476	11.9	<0.001
Dementia	28094	23.6	28094	70.7	0	0	<0.001
Stroke	12472	10.5	4062	10.2	8410	10.6	0.053
Other neurological diseases	3710	3.1	1473	3.7	2237	2.8	<0.001
Diabetes	6411	5.4	2592	6.5	3819	4.8	<0.001
Fracture	4045	3.4	1915	4.8	2130	2.7	<0.001
Multimorbidity	32552	27.3	11539	29.1	21013	26.5	<0.001
Infectious diseases	5780	4.9	2633	6.6	3147	4.0	<0.001

Frequently registered causes of death in the dementia group were dementia (71%), cardiovascular diseases (29%), multimorbidity (29%) and cancer diseases (11%). In the control group, cancer diseases (38%), cardiovascular diseases (33%) and multimorbidity (27%) were the most reported causes of death. In this study, the cause of death was obtained from the SRPC. The SRPC allows for the reporting of multiple diagnoses as underlying conditions contributing to death, which accounts for the cumulative percentage exceeding 100% in Table 2.

### Quality of end-of-life

**Fig. 1:**
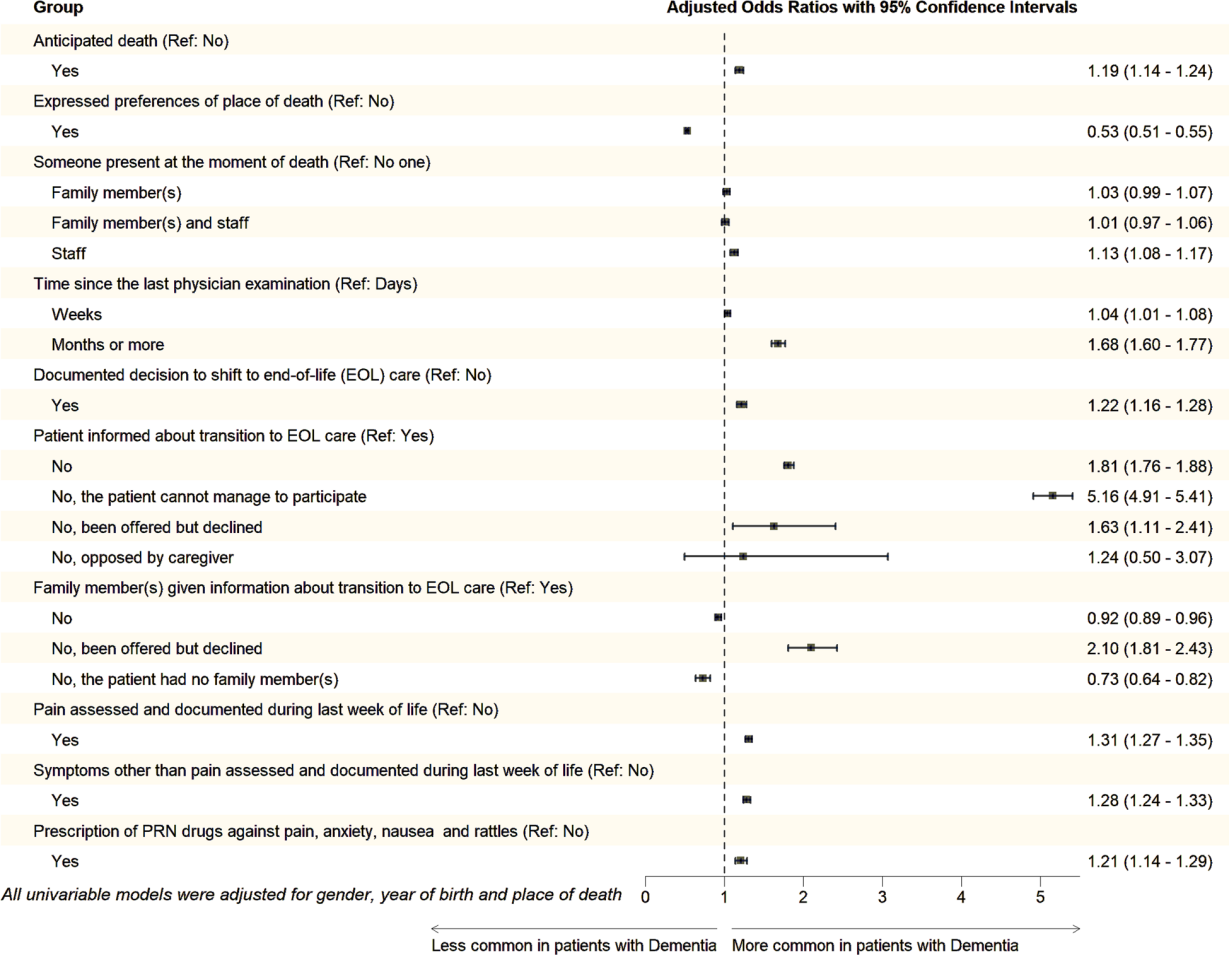
Forest plot showing the odds ratios with 95% confidence intervals from the multiple logistic regression models for the quality indicators in the study sample

**Fig. 2:**
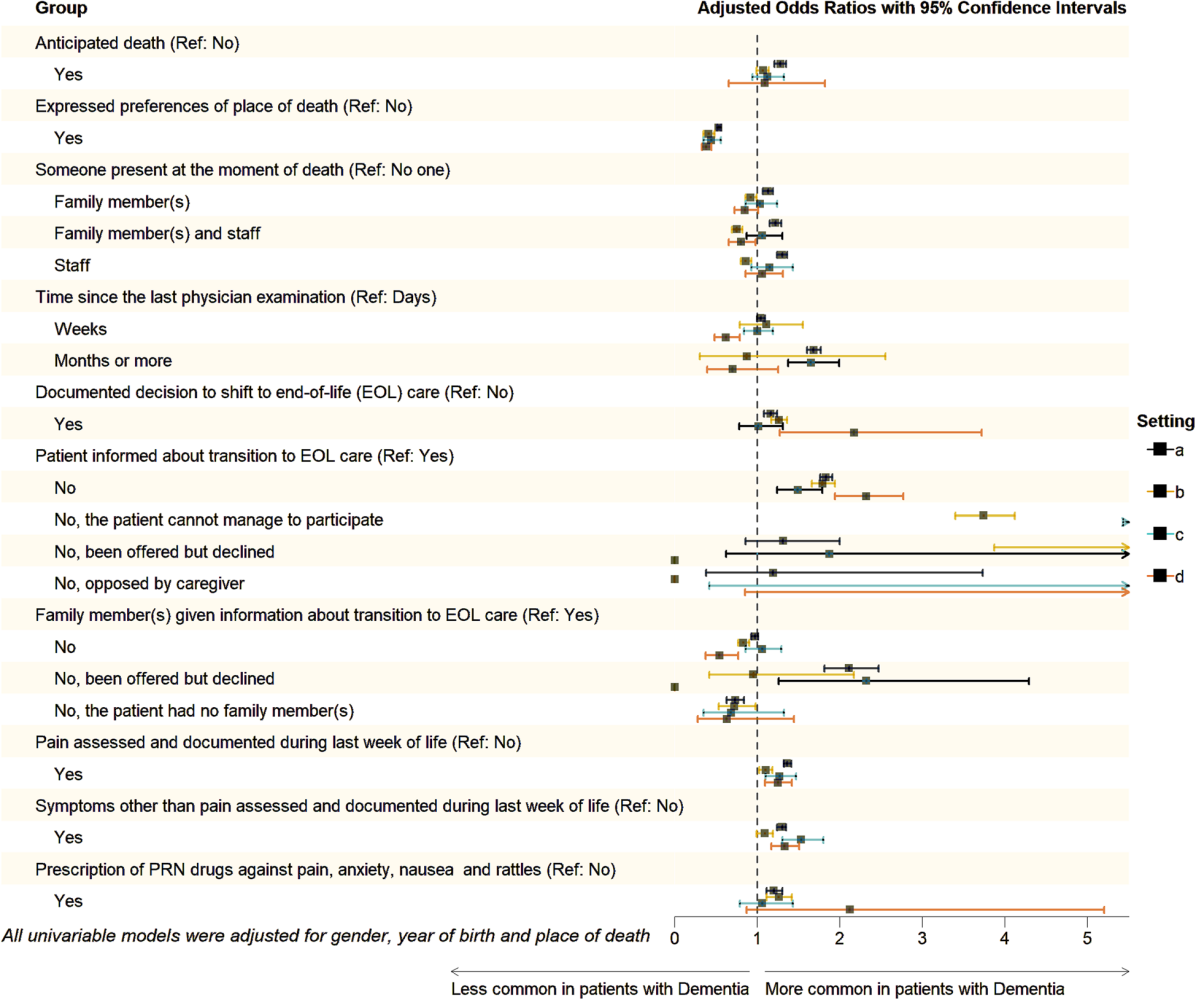
**a**-**d** Forest plots showing the odds ratios with 95% confidence intervals for the quality indicators across four settings: (**a**) nursing homes (NHs), (**b**) hospitals (**c**) ordinary homes and (**d**) specialized palliative care (homecare and in-patient care)

Figure 1 presents a forest plot showing the odds ratios with 95% confidence intervals from the ten adjusted logistic regression models for each quality indicator in the study sample. Fig. 2a-d illustrate the odds ratios (95% confidence intervals) for the quality indicators across four settings: (a) NHs, (b) hospitals (c) ordinary homes and (d) specialized palliative care (homecare and in-patient care). Supplementary Table 1 presents quality indicators from the SRPC for patients with and without dementia. Supplementary Table 2 shows these quality indicators stratified by place of death within the two groups. Supplementary Table 3 presents the odds ratios with 95% confidence intervals along with average marginal effects with 95% confidence intervals from the ten adjusted logistic regression models for each quality indicator.

*Expressed preferences of place of death* were significantly less common in the dementia group with a 47% reduction in odds compared to the control group (Fig. 1). These preferences varied broadly depending on the care setting: only 3–7% of patients and controls in hospitals without specialized palliative care had expressed such preferences, whereas 84–90% in ordinary homes with specialized palliative care had done so (Supplementary Table 2).

*The presence of staff and/or family members at the time of death* was significantly more common in the dementia group compared to the control group (Fig. 1). The likelihood of dying alone varied across care settings. In hospitals, patients with dementia were significantly less likely to have someone present at the time of death compared to controls (Fig. 2b). Among those receiving specialized palliative care at home, only 5% of patients with dementia died alone versus 7% of controls. In NHs, the corresponding figures were 15% and 18%, respectively. The highest proportion of patients dying alone was observed in hospital units without specialized palliative care, where 27% of patients with dementia died alone, compared to 24% of controls (Supplementary Table 2).

In the dementia group, *the time since the last physician examination prior to death* was significantly more likely to be extended, with a 4% higher likelihood for a duration of weeks and a 68% increase in odds for a month or more, compared to the control group (Fig. 1). Conversely, when death occurred in hospitals, extended intervals were significantly less common among individuals in the dementia group, with an 11% increase of odds for weeks but a 13% reduction for a month or more (Fig. 2b).

*Documented decisions to shift to EOL care* were significantly more common in the dementia group, with a 22% increase in odds compared to the controls (Fig. 1). Such documented decisions were performed in approximately 75% of the patients with dementia and 65% of the controls dying in NHs, compared to 55–64% in hospitals without specialized palliative care (Supplementary Table 2). Compared to the control group, patients with dementia had significantly higher odds of not being informed about the transition to EOL care, with an 81% increase. In contrast, the odds of family members not receiving this information were significantly lower in the dementia group, with an 8% reduction (Fig. 1).

*Assessment of pain during the final week of life* was significantly more likely in the dementia group, with a 31% increase in odds compared to the controls (Fig. 1). The rates varied across care settings: in hospitals without specialized palliative care, pain assessment was documented in 19% of the dementia population and 17% of controls, while in specialized palliative care wards, it was 67% and 61%. Across all care settings, *severe pain occurred* significantly less likely in dementia patients than in controls (Supplementary Table 2). *Symptom assessment beyond pain during the final week of life* was significantly more likely in the dementia group, with a 28% increase in odds compared to controls (Fig. 1). *Prescription of injectables* on an as-needed basis against pain, anxiety, nausea and death rattles were significantly more common in the dementia group, with an 21% increase in odds compared to the control group (Fig. 1). In NHs, injectables on an as-needed basis were prescribed significantly more often than in hospitals without specialized palliative care, for both groups (Supplementary Table 2).

Results from a separate analysis excluding the year 2020 (Covid-19 pandemic with a higher number of deaths among older persons) showed no significant differences except for less deaths due to infections (data not shown).

## Discussion

While prior studies have highlighted deficiencies in the quality of palliative care for individuals with dementia, the present study demonstrates that, in several domains, the quality of EOL care, as assessed using quality indicators from the SRPC, is higher among patients with a dementia diagnosis compared to those without.

### Place of death and expressed preferences

In this registry-based study, we found that patients with dementia were significantly less likely to have expressed preferences regarding place of death compared to those without dementia, especially when death occurred in a hospital setting without specialized palliative care. Such preferences were documented for fewer than one-third of individuals with dementia, with only slightly higher proportions in the control group. Consistent with previous research, most individuals with dementia, nearly 80%, died in NHs, reflecting findings that dementia markedly increases the likelihood of dying in a NH [24, 26, 27] and that NHs constitute the dominant place of death for individuals with dementia [24, 25]. However, previous research also highlights that the place of death for individuals with dementia varies internationally, influenced by for example differences in healthcare systems and cultural preferences [24, 41].

In the control group, NHs were the second most common place of death (34%), closely following hospitals (40%), which aligns with prior findings indicating that older individuals often die in NH settings [22, 23]. In our study, nearly one in five individuals in the dementia population died in hospital, which is in line with findings from Lamberg et al., who reported that one in four individuals with advanced dementia residing in NHs are transferred to hospital during the last six months of life [32].

Evidence from studies of place of death [27] and long-term care provision in England [26] suggests that individuals with dementia are more likely than age-matched individuals to spend both the final phase of life and the moment of death in NHs, due to their substantially higher dependency and care needs. Meanwhile, as in many Western countries, hospitals continue to be the most common place of death in England, whereas NHs are the most common place of death in Sweden among individuals aged 65 years and older [42].

### Congruence between preferences and actual place of death

Congruence between preferred and actual place of death is considered a key quality indicator in EOL care [43–45]. Although many patients express a strong preference to die at home, previous research has shown that a significant proportion ultimately die in hospital settings [30, 46]. This study does not address whether such congruence was achieved. Ideally, the quality indicator should address this alignment, rather than the presence of documented expressed preferences. Individuals with dementia often face challenges in articulating such preferences, due to cognitive decline and communication barriers. These preferences may have been discussed earlier in the disease trajectory, underscoring the importance of consistent information transfer throughout the care process. Additionally, this issue is complicated by individual variation in how early, and to what extent, patients wish to engage in EOL care planning [47]. Respecting person-centered preferences, including the degree of involvement in the planning for future care is essential. Given that many individuals with dementia may wish to remain at home at the end of their lives, it is crucial to create the structural and clinical conditions that allow this preference to be realized safely and in a person-centered manner. To support individuals in dying at home, when that is their preference, it is crucial to establish conditions to enable high-quality palliative care in that setting. This aligns with previous research emphasizing the importance of well-resourced, community-based support systems, including homecare services, support for transition from hospital to home, and coordinated care for patients with complex needs [30].

Advance care planning (ACP) is a key strategy for ensuring that EOL care aligns with individual preferences, including the desired place of death and other personally important conditions, while also helping to prevent unnecessary hospital transfers or admissions that may conflict with the patient’s expressed wishes. ACP is a proactive decision-making process involving patients, families, and healthcare providers, aimed at identifying care priorities and planning future care that aligns with the patient’s values and preferences [48, 49]. It is widely recognized as a fundamental component of high-quality palliative care [50, 51]. One specific challenge in ACP for individuals with dementia is the difficulty in communicating EOL needs and wishes due to progressive cognitive impairment and the impaired decision-making capacity over time, related to the progression of the disease [52]. Accordingly, ACP is recommended in the early stages of dementia, when individuals can still express their preferences regarding quality of life and future care before significant cognitive decline occurs [53]. Evidence indicates that early initiation of ACP is associated with improved quality of life and may reduce healthcare utilization [53, 54]. The importance of involving both the patient and their family members in the care planning process have been highlighted [51]. Despite universal recognition of its importance, ACP remains infrequently performed in patients with dementia, and evidence-based recommendations on when and how to perform this complex process are lacking [55].

Recent qualitative research highlights the need to strengthen capacity and consent processes at key EOL decision points [56]. In English NHs, anticipatory prescribing of injectable controlled drugs (ICDs), is often carried out with limited efforts to involve the individual with dementia in line with best-interest principles, raising concerns about self-determination. In addition, the involvement of family members in such decisions tends to be retrospective rather than prospective. Therefore, embedding explicit capacity assessments, prospective family discussions, and scheduled reviews at the time of anticipatory prescribing has been recommended to secure autonomy and improve preference-concordant care [56, 57].

### Presence at time of death

Our study found that individuals with dementia were less likely to die alone compared to controls. However, in hospital settings, the opposite trend was observed - the presence of staff and/or family members at the time of death was less common in the dementia group. Previous research has repeatedly emphasized the association between having someone present at the time of death and the experience of a “good death” [58–60]. However, some studies suggest that dying alone may not be distressing from the patient’s perspective. Instead, the presence of someone at the time of death often holds greater emotional significance for family members and healthcare staff than for the dying individual [61, 62]. Thomson et al. further note that perspectives on dying alone can vary, with some individuals in a NH setting expressing a desire to die alone [63].

### Symptom management in the end-of-life

Our findings show that among patients with dementia, both symptom assessment and documentation during the final week of life as well as prescription of injectables on an as- needed basis (PRN) against pain, anxiety, nausea and death rattles were more common. These patterns persisted across all care settings (Fig. 2a-d) and after adjusting for gender, year of birth and place of death (Fig. 1), suggesting a more active approach to symptom management in EOL care for individuals with dementia. Pain control in EOL is considered a fundamental component of a good death [64]. Observer-rated pain assessment tools have become increasingly common and are effective in identifying the presence of pain. Research indicates that using pain scales improves both the detection of pain and severity assessment in older people with impaired cognition [65]. A recent Swedish registry study found that the occurrence of pain, including severe pain, was less common among patients with dementia compared to those with cancer. On the other hand, when pain was present, it was more often fully relieved in the dementia group [66].

Importantly, our data captures prescriptions, but not administrations. Therefore, we cannot conclude that individuals with dementia actually *received* more injectables than controls. The higher PRN prescribing observed, particularly in NHs, aligns with previous findings showing more frequent PRN prescriptions for pain, nausea and anxiety among individuals with dementia dying in NHs compared with hospitals [4], and may reflect healthcare staff’s preparedness for non-oral routes when swallowing or communication are impaired.

Although individuals with dementia typically receive less aggressive treatment at the end of life, previous studies suggest that symptom management may still be insufficient, particularly in hospital settings [67]. While our study indicates more frequent symptom assessment during the final week of life and the prescription of injectables for symptom relief among patients with dementia compared to the controls, it does not provide information on the actual effectiveness of symptom relief or the patient’s experiences. Furthermore, our data cannot explain why pain assessments and documentation were more frequent in the dementia group than in controls. Possible factors include the use of observational pain tools when verbal self-report is restricted (e.g. Abbey Pain Scale) and routine practices in NHs. However, these should be interpreted cautiously, as the registry does not allow us to separate documentation patterns from underlying clinical needs.

### Challenges and strategies for improving end-of-life care in dementia

EOL care for individuals with dementia presents several challenges related to prognostication and identification of palliative care needs, symptom management, ACP and EOL communication, which require careful consideration, knowledge and rich experience. Strengthening the integration of palliative care, beginning with the early identification of patients who are in or approaching a palliative phase, is a crucial first step. Equally important are improving communication skills, including performing EOL conversations, and providing education for healthcare staff. Organizational changes to increase the competence and the proportion of permanent staff to improve continuity and quality of EOL care in patients with dementia in NH settings have been suggested [68]. Several interventions aiming to enhance ACP performance in individuals with dementia have been conducted, yielding mixed results [69, 70]. A U.S. study using an educational video showed limited impact [69], whereas a U.K. study involving facilitated discussions with family members improved EOL decision clarity and perceived care quality [71].

### Future research directions

To ensure high-quality palliative care for patients with dementia, further quantitative and qualitative research is needed to investigate their specific care needs in advanced stages of the disease. This includes examining how these needs differ from those associated with other terminal conditions and identifying effective strategies for tailoring palliative care to support both patients and their family members. In addition, studies exploring the specific educational needs of healthcare professionals caring for patients with dementia to enhance their competence and confidence in delivering palliative care to this patient group and their families are needed.

### Strengths and limitations

A major strength of this study is its large, nationally representative sample. While previous research is often confined to a single care setting and limited geographical area, our study includes data from all types of care settings across the entire country. This approach is relatively rare and significantly enhances the external validity of our findings. To our knowledge, this is the first study to compare individuals with and without dementia across multiple care settings. In this study, the cause of death was obtained from the SRPC rather than the Cause of Death Registry, which is generally considered to have higher validity. However, the primary focus was on the presence or absence of a dementia diagnosis, rather than to investigate the specific cause of death. Moreover, there is a risk of misclassification, as diagnostic codes and administrative data may be inaccurate or inconsistently applied. A known limitation is that palliative care assessments may be influenced by professional subjectivity, as most death questionnaires are completed by a single staff member (67%) [38], making some degree of individual judgement unavoidable. Another limitation of studies that rely on registry-based data is their inability to capture detailed clinical nuances and patient-reported outcomes, thereby restricting the scope for qualitative insights into healthcare processes better studied through alternative approaches. Finally, although dementia severity is an important covariate, the SveDem registry does not include data on dementia stage at baseline. While baseline MMSE at the time of diagnosis was available, no MMSE data were available closer to the time of death. As a result, dementia severity could not be meaningfully incorporated into the analyses.

## Conclusions

This nationwide registry-based study demonstrates that individuals with dementia receive significantly higher quality in EOL care across several domains, including symptom assessment, prescription of injectables on an as-needed basis and documentation of care decisions to shift to EOL care compared with those without dementia. These findings suggest that NH environments, where most individuals with dementia die in Sweden, may be particularly well suited to deliver good palliative care. However, the markedly lower likelihood of expressed preferences for place of death, especially in hospital settings, emphasizes the importance of proactive care planning initiated at an early stage of the disease. Such planning is essential to prevent care that may not benefit the patient, including potentially avoidable hospital transfers or interventions that conflict with the patient’s own preferences. Strengthening communication processes and ensuring continuity of information throughout the illness trajectory may further support care that is aligned with individual preferences.

Further research is needed to deepen the understanding of palliative care and ACP for individuals with dementia, including their experiences, organizational conditions and the educational needs of family members and healthcare staff. Developing tailored strategies that address these aspects is essential for improving preference-concordant, high-quality EOL care in this population.

## Supplementary Information

Supplementary Material 1. Supplementary table 1: Quality indicators from the Swedish Register of Palliative Care (SRPC) for patients with and without dementia.

Supplementary Material 2. Supplementary table 2: Quality indicators from the Swedish Register of Palliative Care (SRPC) for patients with and without dementia, stratified by place of death within the two groups.

Supplementary Material 3. Supplementary table 3: Supplementary table 3: Odds ratios (ORs) and Average Marginal Effects (AMEs) for quality indicators from the Swedish Register of Palliative Care (SRPC) for patients with and without dementia.

## Data Availability

The datasets used and/or analyzed during the current study are available from the corresponding author on reasonable request.

## Abbreviations

ACP: Advance Care Planning
EOL: End-Of-Life
ICD-10: International Classification of Diseases
ICD: Injectable Controlled Drug
NH: Nursing Home
NICE: National Institute for Health and Care Excellence
NRS: Numeric Rating Scale
PRN: Pro Re Nata
SRPC: Swedish Register of Palliative Care
SveDem: The Swedish registry for cognitive/dementia disorders
UCR: Uppsala Clinical Research Center
VAS: Visual Analogue Scale
