# Supplementary material for: Quality of end-of-life care among individuals with and without dementia: a Swedish registry-based study
Source: BMC Palliat Care. 2026 Mar 7;25:89. doi: 10.1186/s12904-026-02037-9 (PMC13064132; doi:10.1186/s12904-026-02037-9)
Supplement: Supplementary file 1 — Supplementary Material 1. Supplementary table 1: Quality indicators from the Swedish Register of Palliative Care (SRPC) for patients with and without dementia. [file 12904_2026_2037_MOESM1_ESM.docx]

| Quality indicator in the SRPC | Total  n 119 048 | | Dementia group  n 39 712 | | Control group  n 79 336 | | *p-*value |
| --- | --- | --- | --- | --- | --- | --- | --- |
|  | n | % | n | % | n | % |  |
| Anticipated death   - Yes - No - Unknown | 100955  14094  3999 | 84.8  11.8  3.4 | 34607  4122  983 | 87.1  10.4  2.5 | 66348  9972  3016 | 83.6  12.6  3.8 | <0.001 |
| Expressed preferences of place of death   - Yes - No - Unknown | 40610  38346  40092 | 34.1  32.2  33.7 | 11235  13623  14854 | 28.3  34.3  37.4 | 29375  24723  25238 | 37.0  31.2  31.8 | <0.001 |
| Someone present at the moment of death   - No one - Family member(s) - Family member(s) and staff - Staff - Unknown | 22808  37963  20431  34830  3016 | 19.2  31.9  17.2  29.3  2.5 | 6820  11318  6416  14137  1021 | 17.2  28.5  16.2  35.6  2.6 | 15988  26645  14015  20693  1995 | 20.2  33.6  17.7  26.1  2.5 | <0.001 |
| Documented decision to shift to end-of-life (EOL) care   - Yes, classification code - Yes, in free text - No - Unknown/Data missing | 14255  65675  10664  28454 | 12.0  55.2  9.0  23.9 | 5044  24036  3092  7540 | 12.7  60.5  7.8  19.0 | 9211  41639 7572  20914 | 11.6  52.5  9.5  26.4 | <0.001 |
| Patient informed about transition to EOL care   - Yes - No - No, the patient cannot manage to participate - No, been offered but declined - No, opposed by caregiver - Unknown/Data missing | 47873  33216  11805  118  27  26009 | 40.2  27.9  9.9  0.1  0.0  21.8 | 12178  12646  7874  54  8  6952 | 30.7  31.8  19.8  0.1  0.0  17.5 | 35695  20570  3931  64  19  19057 | 45.0  25.9  5.0  0.1  0.0  24.0 | <0.001 |
| Family member(s) given information about transition to EOL care   - Yes - No - No, been offered but declined - No, the patient had no next-of-kin - Unknown/Data missing | 77525  16824  909  1301  22489 | 65.1  14.1  0.8  1.1  18.9 | 26311  6173  613  429  6186 | 66.3  15.5  1.5  1.1  15.6 | 51214  10651  296  872  16303 | 64.6  13.4  0.4  1.1  20.5 | <0.001 |
| Time since the last physician examination   - Day(s) - Week(s) - Month or more - Unknown/Data missing | 69966  22719  9862  16501 | 58.8  19.1  8.3  13.9 | 19039  10155  5550  4968 | 47.9  25.6  14.0  12.5 | 50927  12564  4312  11533 | 64.2  15.8  5.4  14.5 | <0.001 |
| Pain assessed and documented during last week of life   - Yes - No - Unknown/Data missing | 39338  57574  22136 | 33.0  48.4  18.6 | 14881  18712  6119 | 37.5  47.1  15.4 | 24457  38862  16017 | 30.8  49.0  20.2 | <0.001 |
| Severe pain documented last week of life   - No - Yes - Unknown/Data missing | 66440  22061  30547 | 55.8  18.5  25.7 | 24033  7127  8552 | 60.5  17.9  21.5 | 42407  14934  21995 | 53.5  18.8  27.7 | <0.001 |
| Symptoms other than pain assessed and documented during last week of life   - Yes - No - Unknown/Data missing | 22444  70742  25862 | 18.9  59.4  21.7 | 8654  23978  7080 | 21.8  60.4  17.8 | 13790  46764  18782 | 17.4  58.9  23.7 | <0.001 |
| Documented confusion last week of life   - No - Yes - Unknown/Data missing | 65718  24072 29258 | 55.2  20.2  24.6 | 21583  8853  9276 | 54.3  22.3  23.4 | 44135  15219  19982 | 55.6  19.2  25.2 | <0.001 |
| Documented anxiety last week of life   - No - Yes - Unknown/Data missing | 45393  46700  26955 | 38.1  39.2  22.6 | 16552  15387  7773 | 41.7  38.7  19.6 | 28841  31313  19182 | 36.4  39.5  24.2 | <0.001 |
| Prescription of PRN drugs against pain   - Yes - No - Unknown/Data missing | 96342  7616  15090 | 80.9  6.4  12.7 | 33073  2287  4352 | 83.3  5.8  11.0 | 63269  5329  10738 | 79.7  6.7  13.5 | <0.001 |
| Prescription of PRN drugs against anxiety   - Yes - No - Unknown/Data missing | 91425  12134  15510 | 76.8  10.2  13.0 | 32076  3205  4433 | 80.8  8.1  11.2 | 59349  8929  11077 | 74.8  11.2  14.0 | <0.001 |
| Prescription of PRN drugs against nausea   - Yes - No - Unknown/Data missing | 77984  24899  16165 | 65.5  20.9  13.6 | 27268  7798  4646 | 68.7  19.6  11.7 | 50716  17101  11519 | 63.9  21.6  14.5 | <0.001 |
| Prescription of PRN drugs against rattles   - Yes - No - Unknown/Data missing | 92001  11726  15321 | 77.3  9.8  12.9 | 32195  3113  4404 | 81.1  7.8  11.1 | 59806  8613  10917 | 75.4  10.9  13.8 | <0.001 |

**Supplementary table 1.** Quality indicators from the Swedish Register of Palliative Care (SRPC) for patients with and without dementia.
