# Supplementary material for: Quality of end-of-life care among individuals with and without dementia: a Swedish registry-based study
Source: BMC Palliat Care. 2026 Mar 7;25:89. doi: 10.1186/s12904-026-02037-9 (PMC13064132; doi:10.1186/s12904-026-02037-9)
Supplement: Supplementary file 2 — Supplementary Material 2. Supplementary table 2: Quality indicators from the Swedish Register of Palliative Care (SRPC) for patients with and without dementia, stratified by place of death within the two groups. [file 12904_2026_2037_MOESM2_ESM.docx]

| Quality indicator in the SRPC  **Supplementary table 2.** Quality indicators from the Swedish Register of Palliative Care (SRPC) for patients with and without dementia, stratified by place of death within the two groups. | Ordinary home, with specialized palliative home care | | Ordinary home, without specialized palliative home care | | Specialized palliative inpatient care | | Inpatient care, not specialized palliative care | | Nursing home | | *p(dementia)* | *p(control)* |
| --- | --- | --- | --- | --- | --- | --- | --- | --- | --- | --- | --- | --- |
|  | Dementia  group  n (%) | Control group  n (%) | Dementia group  n (%) | Control group  n (%) | Dementia group  n (%) | Control group  n (%) | Dementia group  n (%) | Control group  n (%) | Dementia group  n (%) | Control group  n (%) |  |  |
| Anticipated death  Yes  No  Unknown | 261 (97.8)  6 (2.2)  0 (0.0) | 4590 (96.9)  140 (3.0)  6 (0.1) | 888 (81.1)  187 (17.1)  20 (1.8) | 4835 (78.7)  1139 (18.5)  171 (2.8) | 839 (98.5)  10 (1.2)  3 (0.4) | 9181 (98.9)  76 (0.8)  36 (0.4) | 5372 (76.7)  1170 (16.7)  461 (6.6) | 24155 (75.6)  5596 (17.5)  2199 (6.9) | 27247 (89.3)  2749 (9.0)  499 (1.6) | 23587 (86.7)  3021 (11.1)  604 (2.2) | *<0.001* | *<0.001* |
| Expressed preferences of place of death  Yes  No  Unknown | 225 (84.3)  16 (6.0)  26 (9.7) | 4245 (89.6)  102 (2.2)  389 (8.2) | 660 (60.3)  100 (9.1)  335 (30.6) | 4341 (70.6)  291 (4.7)  1513 (24.6) | 339 (39.8)  305 (35.8)  208 (24.4) | 5735 (61.7)  2016 (21.7)  1542 (16.6) | 193 (2.8)  3372 (48.2)  3438 (49.1) | 2173 (6.8)  15710 (49.2)  14067 (44.0) | 9818 (32.2)  9830 (32.2)  10847 (35.6) | 12881 (47.3)  6604 (24.3)  7727 (28.4) | *<0.001* | *<0.001* |
| Someone present at the moment of death  No one  Family member(s)  Family member(s) and staff  Staff  Unknown | 14 (5.2)  174 (65.2)  59 (22.1)  20 (7.5)  0 (0.0) | 329 (6.9)  3133 (66.2)  942 (19.9)  306 (6.5)  26 (0.5) | 200 (18.3)  424 (38.7)  245 (22.4)  207 (18.9)  19 (1.7) | 1166 (19.0)  2411 (39.2)  1344 (21.9)  1040 (16.9)  184 (3.0) | 189 (22.2)  358 (42.0)  137 (16.1)  159 (18.7)  9 (1.1) | 1920 (20.7)  3917 (42.2)  1811 (19.5)  1586 (17.1)  59 (0.6) | 1870 (26.7)  2119 (30.3)  1032 (14.7)  1635 (23.3)  347 (5.0) | 7755 (24.3)  9573 (30.0)  5629 (17.6)  7790 (24.4)  1203 (3.8) | 4547 (14.9)  8243 (27.0)  4943 (16.2)  12116 (39.7)  646 (2.1) | 4818 (17.7)  7611 (28.0)  4289 (15.8)  9971 (36.6)  523 (1.9) | *<0.001* | *<0.001* |
| Time since the last physician examination  Day(s)  Week(s)  Month or more  Unknown | 182 (68.2)  62 (23.2)  12 (4.5)  11 (4.1) | 3149 (66.5)  1228 (25.9)  192 (4.1)  167 (3.5) | 269 (24.6)  329 (30.0)  276 (25.2)  221 (20.2) | 1695 (27.6)  2077 (33.8)  1050 (17.1)  1323 (21.5) | 829 (97.3)  10 (1.2)  0 (0.0)  13 (1.5) | 8989 (96.7)  156 (1.7)  10 (0.1)  138 (1.5) | 5626 (80.3)  42 (0.6)  4 (0.1)  1331 (19.0) | 25496 (79.8)  173 (0.5)  21 (0.1)  6260 (19.6) | 12133 (39.8)  9712 (31.8)  5258 (17.2)  3392 (11.1) | 11598 (42.6)  8930 (32.8)  3039 (11.2)  3645 (13.4) | *<0.001* | *<0.001* |
| Documented decision to shift to end-of-life (EOL) care  Yes, classification code  Yes, in free text  No  Unknown | 68 (25.5)  173 (64.8)  3 (1.1)  23 (8.6) | 974 (20.6)  2797 (59.1)  133 (2.8)  832 (17.6) | 170 (15.5)  588 (53.7)  74 (6.8)  263 (24.0) | 790 (12.9)  3026 (49.2)  375 (6.1)  1954 (31.8) | 270 (31.7)  525 (61.6)  11 (1.3)  46 (5.4) | 2369 (25.5)  5531 (59.5)  206 (2.2)  1187 (12.8) | 368 (5.3)  4082 (58.3)  1003 (14.3)  1550 (22.1) | 1665 (5.2)  15968 (50.0)  4958 (15.5)  9359 (29.3) | 4168 (13.7)  18668 (61.2)  2001 (6.6)  5658 (18.6) | 3413 (12.5)  14317 (52.6)  1900 (7.0)  7582 (27.9) | *<0.001* | *<0.001* |
| Patient informed about transition to EOL care  Yes  No  No, the patient cannot manage to participate  No, been offered but declined  No, opposed by caregiver  Unknown | 178 (66.7)  36 (13.5)  36 (13.5)  0 (0.0)  1 (0.4)  16 (6.0) | 4011 (84.7)  372 (7.9)  51 (1.1)  3 (0.1)  1 (0.0)  298 (6.3) | 550 (50.2)  183 (16.7)  94 (8.6)  4 (0.4)  1 (0.1)  263 (24.0) | 3630 (59.1)  806 (13.1)  84 (1.4)  14 (0.2)  1 (0.0)  1610 (26.2) | 500 (58.7)  138 (16.2)  153 (18.0)  0 (0.0)  0 (0.0)  61 (7.2) | 7538 (81.1)  871 (9.4)  245 (2.6)  3 (0.0)  1 (0.0)  635 (6.8) | 1053 (15.0)  2718 (38.8)  1153 (16.5)  5 (0.1)  0 (0.0)  2074 (29.6) | 7725 (24.2)  11200 (35.1)  2258 (7.1)  1 (0.0)  10 (0.0)  10756 (33.7) | 9897 (32.5)  9571 (31.4)  6438 (21.1)  45 (0.1)  6 (0.0)  4538 (14.9) | 12791 (47.0)  7321 (26.9)  1293 (4.8)  43 (0.2)  6 (0.0)  5758 (21.2) | *<0.001* | *<0.001* |
| Family member(s) given information about transition to EOL care  Yes  No  No, been offered but declined  No, the patient had no next-of-kin  Unknown | 248 (92.9)  9 (3.4)  0 (0.0)  1 (0.4)  9 (3.4) | 4162 (87.9)  257 (5.4)  3 (0.1)  30 (0.6)  284 (6.0) | 688 (62.8)  138 (12.6)  15 (1.4)  10 (0.9)  244 (22.3) | 3650 (59.4)  687 (11.2)  34 (0.6)  78 (1.3)  1696 (27.6) | 795 (93.3)  22 (2.6)  0 (0.0)  5 (0.6)  30 (3.5) | 8338 (89.7)  426 (4.6)  7 (0.1)  82 (0.9)  440 (4.7) | 4304 (61.5)  909 (13.0)  7 (0.1)  50 (0.7)  1733 (24.7) | 18222 (57.0)  4584 (14.3)  31 (0.1)  281 (0.9)  8832 (27.6) | 20276 (66.5)  5095 (16.7)  591 (1.9)  363 (1.2)  4170 (13.7) | 16842 (61.9)  4697 (17.3)  221 (0.8)  401 (1.5)  5051 (18.6) | *<0.001* | *<0.001* |
| Pain assessed and documented during last week of life  Yes  No  Unknown | 158 (59.2)  101 (37.8)  8 (3.0) | 2751 (58.1)  1713 (36.2)  272 (5.7) | 391 (35.7)  473 (43.2)  231 (21.1) | 1851 (30.1)  2820 (45.9)  1474 (24.0) | 568 (66.7)  246 (28.9)  38 (4.5) | 5687 (61.2)  3204 (34.5)  402 (4.3) | 1326 (18.9)  3708 (52.9)  1969 (28.1) | 5463 (17.1)  16834 (52.7)  9653 (30.2) | 12438 (40.8)  14184 (46.5)  3873 (12.7) | 8705 (32.0)  14291 (52.5)  4216 (15.5) | *<0.001* | *<0.001* |
| Severe pain documented last week of life  No  Yes  Unknown | 189 (70.8)  57 (21.3)  21 (7.9) | 3082 (65.1)  1176 (24.8)  478 (10.1) | 628 (57.4)  189 (17.3)  278 (25.4) | 3224 (52.5)  1207 (19.6)  1714 (27.9) | 600 (70.4)  186 (21.8)  66 (7.7) | 5852 (63.0)  2598 (28.0)  843 (9.1) | 3135 (44.8)  779 (11.1)  3089 (44.1) | 14110 (44.2)  4172 (13.1)  13668 (42.8) | 19481 (63.9)  5916 (19.4)  5098 (16.7) | 16139 (59.3)  5781 (21.2)  5292 (19.4) | *<0.001* | *<0.001* |
| Symptoms other than pain assessed and documented during last week of life  Yes  No  Unknown | 94 (35.2)  160 (59.9)  13 (4.9) | 1528 (32.3)  2849 (60.2)  359 (7.6) | 254 (23.2)  590 (53.9)  251 (22.9) | 1007 (16.4)  3552 (57.8)  1586 (25.8) | 372 (43.7)  436 (51.2)  44 (5.2) | 3349 (36.0)  5342 (57.5)  602 (6.5) | 705 (10.1)  3843 (54.9)  2455 (35.1) | 2919 (9.1)  17403 (54.5)  11628 (36.4) | 7229 (23.7)  18949 (62.1)  4317 (14.2) | 4987 (18.3)  17618 (64.7)  4607 (16.9) | *<0.001* | *<0.001* |
| Prescription of PRN drugs against pain  Yes  No  Unknown | 256 (95.9)  5 (1.9)  6 (2.2) | 4480 (94.6)  114 (2.4)  142 (3.0) | 837 (76.4)  67 (6.1)  191 (17.4) | 4559 (74.2)  376 (6.1)  1210 (19.7) | 833 (97.8)  8 (0.9)  11 (1.3) | 9139 (98.3)  72 (0.8)  82 (0.9) | 5157 (73.6)  532 (7.6)  1314 (18.8) | 22792 (71.3)  2975 (9.3)  6183 (19.4) | 25990 (85.2)  1675 (5.5)  2830 (9.3) | 22299 (81.9)  1792 (6.6)  3121 (11.5) | *<0.001* | *<0.001* |
| Prescription of PRN drugs against anxiety  Yes  No  Unknown | 256 (95.9)  5 (1.9)  6 (2.2) | 4397 (92.8)  194 (4.1)  145 (3.1) | 823 (75.2)  79 (7.2)  193 (17.6) | 4401 (71.6)  518 (8.4)  1226 (20.0) | 833 (97.8)  7 (0.8)  12 (1.4) | 9058 (97.5)  148 (1.6)  87 (0.9) | 4823 (68.9)  835 (11.9)  1345 (19.2) | 20166 (63.1)  5341 (16.7)  6443 (20.2) | 25341 (83.1)  2277 (7.5)  2877 (9.4) | 21327 (78.4)  2709 (10.0)  3176 (11.7) | *<0.001* | *<0.001* |
| Prescription of PRN drugs against nausea  Yes  No  Unknown | 245 (91.8)  14 (5.2)  8 (3.0) | 4246 (89.7)  337 (7.1)  153 (3.2) | 741 (67.7)  155 (14.2)  199 (18.2) | 4014 (65.3)  883 (14.4)  1248 (20.3) | 813 (95.4)  27 (3.2)  12 (1.4) | 8864 (95.4)  332 (3.6)  97 (1.0) | 3564 (50.9)  2018 (28.8)  1421 (20.3) | 14923 (46.7)  10276 (32.2)  6751 (21.1) | 21905 (71.8)  5584 (18.3)  3006 (9.9) | 18669 (68.6)  5273 (19.4)  3270 (12.0) | *<0.001* | *<0.001* |
| Prescription of PRN drugs against rattles  Yes  No  Unknown | 252 (94.4)  7 (2.6)  8 (3.0) | 4334 (91.5)  255 (5.4)  147 (3.1) | 824 (75.3)  80 (7.3)  191 (17.4) | 4414 (71.8)  506 (8.2)  1225 (19.9) | 826 (96.9)  15 (1.8)  11 (1.3) | 8960 (96.4)  247 (2.7)  86 (0.9) | 4865 (69.5)  809 (11.6)  1329 (19.0) | 20489 (64.1)  5155 (16.1)  6306 (19.7) | 25428 (83.4)  2202 (7.2)  2865 (9.4) | 21609 (79.4)  2450 (9.0)  3153 (11.6) | *<0.001* | *<0.001* |
