# Supplementary material for: Quality of end-of-life care among individuals with and without dementia: a Swedish registry-based study
Source: BMC Palliat Care. 2026 Mar 7;25:89. doi: 10.1186/s12904-026-02037-9 (PMC13064132; doi:10.1186/s12904-026-02037-9)
Supplement: Supplementary file 3 — Supplementary Material 3. Supplementary table 3: Supplementary table 3: Odds ratios (ORs) and Average Marginal Effects (AMEs) for quality indicators from the Swedish Register of Palliative Care (SRPC) for patients with and without dementia. [file 12904_2026_2037_MOESM3_ESM.docx]

**Supplementary table 3.** Quality indicators from the Swedish Register of Palliative Care (SRPC) for patients with and without dementia.

| **Quality Indicator** | **OR*** | **95% CI Lower** | **95% CI Upper** | **AME*** | **95% CI Lower** | **95% CI Upper** |  |  |
| --- | --- | --- | --- | --- | --- | --- | --- | --- |
| ***Anticipated death*** |  |  |  |  |  |  |  |  |
| No | Ref |  |  | Ref |  |  |  |  |
| Yes | 1.19 | 1.14 | 1.24 | 3.1% | 2.4% | 3.9% |  |  |
| Unknown | 0.99 | 0.90 | 1.08 | -0.3% | -1.8% | 1.3% |  |  |
| ***Expressed preferences of place of death*** |  |  |  |  |  |  |  |  |
| No | Ref |  |  | Ref |  |  |  |  |
| Yes | 0.53 | 0.51 | 0.55 | -11.2% | -11.8% | -10.6% |  |  |
| Unknown | 0.97 | 0.94 | 1.00 | -0.5% | -1.1% | 0.1% |  |  |
| ***Someone present at the moment of death*** |  |  |  |  |  |  |  |  |
| No one | Ref |  |  | Ref |  |  |  |  |
| Family member(s) | 1.03 | 0.99 | 1.07 | 0.4% | -0.3% | 1.2% |  |  |
| Family member(s) and staff | 1.01 | 0.97 | 1.06 | 0.2% | -0.6% | 1.0% |  |  |
| Staff | 1.13 | 1.08 | 1.17 | 2.2% | 1.5% | 2.9% |  |  |
| Unknown | 1.24 | 1.13 | 1.35 | 3.9% | 2.3% | 5.6% |  |  |
| ***Time since the last physician examination*** |  |  |  |  |  |  |  |  |
| Day(s) | Ref |  |  | Ref |  |  |  |  |
| Week(s) | 1.04 | 1.01 | 1.08 | 0.8% | 0.1% | 1.5% |  |  |
| Month or more | 1.68 | 1.60 | 1.77 | 10.0% | 9.0% | 11.0% |  |  |
| Unknown | 0.92 | 0.89 | 0.96 | -1.4% | -2.2% | -0.7% |  |  |
| ***Documented decision to shift to end-of-life (EOL) care*** |  |  |  |  |  |  |  |  |
| No | Ref |  |  | Ref |  |  |  |  |
| Yes | 1.22 | 1.16 | 1.28 | 3.6% | 2.7% | 4.5% |  |  |
| Unknown | 0.74 | 0.70 | 0.78 | -5.2% | -6.1% | -4.2% |  |  |
| ***Patient informed about transition to EOL care*** |  |  |  |  |  |  |  |  |
| Yes | Ref |  |  | Ref |  |  |  |  |
| No | 1.81 | 1.76 | 1.88 | 10.7% | 10.1% | 11.4% |  |  |
| No, the patient cannot manage to participate | 5.16 | 4.91 | 5.41 | 31.3% | 30.4% | 32.2% |  |  |
| No, been offered but declined | 1.63 | 1.11 | 2.41 | 8.7% | 1.4% | 16.0% |  |  |
| No, opposed by caregiver | 1.24 | 0.50 | 3.07 | 3.7% | -12.4% | 19.7% |  |  |
| Unknown | 1.18 | 1.13 | 1.22 | 2.8% | 2.1% | 3.4% |  |  |
| ***Family member(s) given information about transition to EOL care*** |  |  |  |  |  |  |  |  |
| Yes | Ref |  |  | Ref |  |  |  |  |
| No | 0.92 | 0.89 | 0.96 | -1.5% | -2.2% | -0.8% |  |  |
| No, been offered but declined | 2.10 | 1.81 | 2.43 | 14.4% | 11.5% | 17.3% |  |  |
| No, the patient had no family members | 0.73 | 0.64 | 0.82 | -5.8% | -8.0% | -3.6% |  |  |
| Unknown | 0.74 | 0.71 | 0.77 | -5.5% | -6.1% | -4.8% |  |  |
| ***Pain assessed and documented during last week of life*** |  |  |  |  |  |  |  |  |
| No | Ref |  |  | Ref |  |  |  |  |
| Yes | 1.31 | 1.27 | 1.35 | 5.0% | 4.4% | 5.5% |  |  |
| Unknown | 0.92 | 0.88 | 0.95 | -1.6% | -2.3% | -0.9% |  |  |
| ***Symptoms other than pain assessed and documented during last week of life*** |  |  |  |  |  |  |  |  |
| No | Ref |  |  | Ref |  |  |  |  |
| Yes | 1.28 | 1.24 | 1.33 | 4.6% | 3.9% | 5.3% |  |  |
| Unknown | 0.90 | 0.87 | 0.93 | -1.9% | -2.6% | -1.3% |  |  |
| ***Prescription of PRN drugs against pain, anxiety, nausea and rattles*** |  |  |  |  |  |  |  |  |
| No | Ref |  |  | Ref |  |  |  |  |
| Yes | 1.21 | 1.14 | 1.29 | 3.5% | 2.4% | 4.6% |  |  |
| Unknown | 1.03 | 0.95 | 1.10 | 0.4% | -0.9% | 1.7% |  |  |

*All models were adjusted for gender, year of birth and place of death

OR = Odds Ratio; AME = Average Marginal Effect; 95% CI = 95% Confidence Interval

Significant OR values above 1 indicate that it is more common in patients with dementia.
